# The effect of cell surface components on adhesion ability of *Lactobacillus rhamnosus*

**DOI:** 10.1007/s10482-014-0245-x

**Published:** 2014-08-05

**Authors:** Magdalena Polak-Berecka, Adam Waśko, Roman Paduch, Tomasz Skrzypek, Anna Sroka-Bartnicka

**Affiliations:** 1 Department of Biotechnology, Human Nutrition and Science of Food Commodities, University of Life Sciences in Lublin, Skromna 8, 20-704 Lublin, Poland; 2 Institute of Agrophysics of Polish Academy of Sciences, Doświadczalna 4, 20-290 Lublin, Poland; 3 Department of Virology and Immunology, Institute of Microbiology and Biotechnology, Maria Curie-Skłodowska University, Lublin, Poland; 4 Laboratory of Confocal and Electron Microscopy, Interdisciplinary Research Center, John Paul II Catholic University of Lublin, Lublin, Poland; 5 Laboratory of Medicinal Chemistry and Neuroengineering, Medical University of Lublin, Lublin, Poland

**Keywords:** Adhesion, Exopolysaccharides, Hydrophobicity, Surface proteins, *Lactobacillus rhamnosus*

## Abstract

The aim of this study was to analyze the cell envelope components and surface properties of two phenotypes of *Lactobacillus rhamnosus* isolated from the human gastrointestinal tract. The ability of the bacteria to adhere to human intestinal cells and to aggregate with other bacteria was determined. *L. rhamnosus* strains E/N and PEN differed with regard to the presence of exopolysaccharides (EPS) and specific surface proteins. Transmission electron microscopy showed differences in the structure of the outer cell surface of the strains tested. Bacterial surface properties were analyzed by Fourier transform infrared spectroscopy, fatty acid methyl esters and hydrophobicity assays. Aggregation capacity and adhesion of the tested strains to the human colon adenocarcinoma cell line HT29 was determined. The results indicated a high adhesion and aggregation ability of *L. rhamnosus* PEN, which possessed specific surface proteins, had a unique fatty acid content, and did not synthesize EPS. Adherence of *L. rhamnosus* was dependent on specific interactions and was promoted by surface proteins (42–114 kDa) and specific fatty acids. Polysaccharides likely hindered bacterial adhesion and aggregation by masking protein receptors. This study provides information on the cell envelope constituents of lactobacilli that influence bacterial aggregation and adhesion to intestinal cells. This knowledge will help to understand better their specific contribution in commensal–host interactions and adaptation to this ecological niche.

## Introduction

*Lactobacillus* strains that inhabit the human gastrointestinal tract (GIT) contribute to the autochthonous microflora that colonize the intestine stably throughout the host’s lifetime (Reuter 2001). Some lactobacilli are known as probiotic microorganisms with a wide range of health promoting effects. The protective role of lactobacilli is probably the result of a combined effect of acidification of the local environment, and competitive exclusion. Coaggregation abilities may form a barrier that prevents colonization by pathogenic microorganisms. The binding of probiotic bacteria to intestinal cells is considered a prerequisite for pathogen exclusion and immunomodulation (Turpin et al. 2012).

The surface characteristics of lactobacilli contribute in several ways to their interactions with the host GIT and the gut microbiota, affecting their survival, adherence to the host tissue, and interactions with their own species and with other bacteria (Lebeer et al. 2008; Kleerebezem et al. 2010). Nonspecific interactions of bacteria with their environment are governed by the physicochemical properties of the cell envelope, particularly its outer constituents (Schär-Zammaretti and Ubbink 2003). The adhesion behavior of microbial cells has been shown to depend on the balance of electrostatic and van der Waals interactions and on the hydrophobic character of the surfaces (Boonaert and Rouxhet 2000). Hydrophobicity plays a key role in the first contact between a bacterial cell and mucous or epithelial cells (Schillinger et al. 2005). The conformation of surface polymers is of major importance for the overall physicochemical properties of bacteria. However, the relationship between the structural organization of the cell surface constituents and physicochemical interactions of bacteria with their environment is still largely an open question. The adhesion ability of lactobacilli has also been linked with specific interactions involving the recognition of a specific site or ligand by a receptor on the bacterial surface (Schär-Zammaretti and Ubbink 2003).

Bacteria from the genus *Lactobacillus* could well serve as model systems for the study of structure–property relations of the bacterial cell envelope (Schär-Zammaretti and Ubbink 2003). The peptidoglycan layer of the cell wall of lactic acid bacteria is covered by (lipo) teichoic acids, surface proteins, and polysaccharides. Lipoteichoic acids with their strongly acidic phosphate groups have a pronounced polyelectrolyte character (Sengupta et al. 2013). Surface proteins, especially S-layer proteins, in many *Lactobacillus* species are noncovalently bound to the cell wall; they are highly basic with a high isoelectric point. Surface proteins are expected to have appreciable effects on the properties of the cell wall of many *Lactobacillus* strains (Schär-Zammaretti and Ubbink 2003).

The polysaccharides associated with the bacterial cell envelope (CPS) and the extracellular polysaccharides (EPS) of lactic acid bacteria are either neutral or acidic (Ricciardi and Clementi 2000; Sengupta et al. 2013). Because of their abundance and their presence on the outer surface of the cell envelope, EPS and CPS determine the surface properties of microorganisms to a large extent (Boonaert and Rouxhet 2000; Schär-Zammaretti and Ubbink 2003). Accumulating evidence suggests that EPS can have an important influence on bacterial aggregation, biofilm formation, adhesion, and survival (Walter et al. 2008; Lebeer et al. 2009). Górska-Frączek et al. (2011) have reported that the polysaccharide structure may affect bacterial adhesion to mucus. Other authors have claimed that, in some cases, the EPS envelope covering the producing strains hinders bacterial adhesion (Nikolic et al. 2012). Overall, however, the role of bacterial EPS in the colonization of intestinal mucosa is still unclear. Understanding of the factors that influence the surface properties of lactobacilli will certainly support selection and evaluation of probiotic strains. This premise motivated us to undertake the present study on the physicochemical cell surface properties of *L. rhamnosus* strains differing with respect to their EPS synthesis capacity, cell surface proteins and fatty acid components. To our knowledge, this is the first study that tries to relate the physicochemical properties of two different bacterial phenotypes isolated from the same environment to their functional aspects as probiotics, such as adhesion to the human GIT.

The aim of this study was to analyze the cell envelope components and to determine bacterial surface properties of two *L. rhamnosus* phenotypes isolated from the human GIT. An attempt was made to identify the cell envelope components responsible for the adhesion to intestinal cells and aggregation with other bacterial cells.

## Materials and methods

### Bacterial strains and culture conditions

The strains isolated from the human GIT, *L. rhamnosus* E/N and PEN, were obtained from Biomed Serum and Vaccine Production Plant Ltd. in Lublin (Poland). They are components of Lakcid^®^, a pharmaceutical product containing viable probiotic bacteria. The strains are deposited at the Institute of Biochemistry and Biophysics of the Polish Academy of Sciences under numbers 2593 (*L. rhamnosus* PEN) and 2594 (*L. rhamnosus* E/N). The bacteria were stored at −80 °C in MRS medium with addition of 20 % (v/v) glycerol. The strains were revitalized in MRS broth at 37 °C for 24 h before use. For experiments, the bacteria were cultivated in MRS medium at 37 °C for 18 h and inoculated as a 2.5 % (v/v) overnight culture in the same medium.

Pathogenic bacteria used in the coaggregation experiment came from the collection of the Department of Biotechnology, Human Nutrition and Food Commodities, University of Life Sciences in Lublin. The following cultures were used: Gram-positive *Staphylococcus aureus* and Gram-negative *Salmonella anatum*. The strains were stored at −80 °C in 20 % glycerol. The bacteria were grown overnight in nutrient broth at 37 °C.

### Detection of exopolysaccharide (EPS) producing strains

The ability of the bacteria to produce EPS was determined by visual appearance (Ruas-Madiedo and De Los Reyes-Gavilán 2005; Begovic et al. 2010). The indicator of the presence of EPS was forming a weak pellet after centrifugation. Ropiness of colonies on MRS agar was tested with a loop to observe the formation of slime, which indicates EPS production. The mucoid colonies were not able to produce strands when extended with an inoculation loop. Slime production was also demonstrated by the qualitative method of Christensen et al. (1985) using a semi-quantitative scale (− to +++).

### Transmission electron microscopy (TEM)

To observe the polysaccharides produced by *L. rhamnosus* on the cell surface, cell surface structures were visualized by negative staining using TEM. *L. rhamnosus* cells were collected by centrifugation, fixed in 2 % glutaraldehyde in 100 mM phosphate buffer at pH 7.2 (4 °C, 2 h), post-fixed in 2 % osmium tetroxide in the same buffer, rinsed three times with the buffer, dehydrated in an ethanol series: 30, 50, 75, 96 % (15 min each), and finally washed three times in 100 % ethanol (15 min each change at room temperature). The samples were then infiltrated with agar low viscosity resin (R1078) according to the following protocol: resin:ethanol (1:1) 60 min; resin:ethanol (1:2) 60 min; 100 % resin 60 min; 100 % resin polymerization at 70 °C 2 days. The specimens were cut into 200 nm sections and examined at an acceleration voltage of 80 kV in TEM (Libra 120, Zeiss).

### Detection of cell wall-associated proteins

Bacterial cultures were adjusted to the same optical density OD_600_ of 0.5, and 1.0 ml of each culture was centrifuged at 15,000×*g* and 4 °C for 15 min. The cell pellets were washed twice with deionised water and re-suspended in 30 µl SDS-PAGE sample buffer. The suspensions were heated for 5 min at 100 °C and centrifuged as above. Ten microlitres of the supernatants was checked for the presence of whole cell proteins by SDS-PAGE as described by Laemmli (1970).

For isolation of non-covalently bound surface proteins, the bacterial biomass from 150 ml cultures grown in MRS medium for 2 days at 37 °C was harvested by centrifugation (9,000×*g*, 4 °C) and washed twice with PBS buffer. 0.2 g of wet cells was extracted with 1 ml 5 mol l^−1^ LiCl (Lortal et al. 1992) for 2 h on a shaker (200 rpm). Cell pellet was removed by centrifugation (9,000×*g*, 4 °C) and supernatant was dialyzed against deionized water at 4 °C. Finally, the isolated cell envelope proteins were lyophilized. Samples were frozen at −80 °C for one hour and then freeze-dried in a freeze-dryer (Labconco, Kansas City, USA) at −50 °C and 0.024 mBar for 18 h. The dried proteins were stored at −20 °C.

1 mg of isolated proteins was re-suspended in 1 ml of SDS-PAGE sample buffer. The suspensions were heated for 5 min at 100 °C and centrifuged (9,000×*g*, 4 °C). Ten microliters of the supernatants (equal to 10 μg of protein) were checked for the presence of surface proteins by SDS-PAGE, as described by Laemmli (1970). The gels were photographed using the Gel-Doc documentation system and analyzed by Quantity One Analysis Software (Biorad, USA).

### Surface analysis by FT-IR

FT-IR spectra were collected on a Nicolet 8700 FT-IR spectrometer (Thermo Scientific, Waltham, MA, USA) equipped with a KBr beam-splitter and a Mercury Cadmium Telluride MCT/A detector in transreflecting mode. The spectra were recorded over the range of 4,000–650 cm^−1^. Each spectrum represented an average of 120 scans and was apodized with a Happ-Genzel function; the number of scan points was 8,480. The spectral resolution was 8 cm^−1^. Each sample was measured five times. Each spectrum was baseline corrected and then the spectra were normalized and averaged. The averaged spectra were normalized to minimize differences due to sample size and the baseline was corrected in all spectral regions. Assignment of the functional groups of the FT-IR spectra was made according to Naumann et al. (1991). To prepare samples, freeze-dried bacteria were ground on an aluminum-coated microscope glass slide. The background spectrum was recorded for every sample. FTIR spectra were measured and analyzed using Atlus microscopy Software for OMNIC-8.

### Fatty acid methyl ester (FAME) analysis

Overnight cultures were centrifuged and the pellets were washed three times with Mili-Q water. Bacterial cells were lyophilized at −50 °C, 0.024 mBar for 18 h (Labconco, USA). Next, 4 ml of methanol:chloroform (2 : 1, v/v) was added to 0.5 ml of suspensions containing approx. 60–70 mg of lyophilized cell biomass in Milli Q water. The samples were kept at room temperature for 1 h with vortexing, and left at 4 °C for 1 h. In order to separate the system into two phases, 1 ml of water was added into each tube and centrifuged (2,000×*g*, 20 min, 4 °C). 2 ml of the chloroform-rich (bottom) phase containing cellular lipids was evaporated under nitrogen. The lipid residue was immediately dissolved with 1 ml of anhydrous hexane. FAMEs were synthesized using 0.4 ml of 2 M KOH/methanol as a transesterifying agent. The reaction was performed at room temperature for 2 h. The FAMEs were analyzed using a gas chromatograph (Shimadzu GC2010, Japan) interfaced with a quadrupole mass spectrometer (Shimadzu QP2010Plus, Japan) and autosampler (Shimadzu AOC-20i, Japan). The injector worked in a split (1:20) mode; the injector temperature was 250 °C. A VF-5MS capillary column (CP8944, 30 m, 0.25 mm i.d., 0.25 µm film thickness; Varian, USA) was used for separation. The oven temperature was programmed in three stages. i.e. from 40 °C (held for 4 min) to 160 °C, in increments of 30 °C min^−1^; from 160 °C to 205 °C, at 2 °C min^−1^; and finally from 205 to 290 °C (for 5 min), at 10 °C min^−1^. Helium was used as the carrier gas at a flow rate of 1.0 ml min^−1^. The mass spectrometer was operated in the scan mode with electron ionization of 70 eV. The interface temperature was set to 240 °C and the ion source temperature was adjusted to 200 °C. 1-bromotetradecane was used as an internal standard for quantification of fatty acid methyl esters. FAME identification was performed on the basis of retention time of the FAME standard (Supelco 37 component FAME Mix, Supelco, USA), and the mass spectrum was compared to reference spectra (NIST 5.0).

### Determination of bacterial hydrophobicity

Cell surface hydrophobicity was determined by the Salt Aggregation Test (SAT), as previously described (Lindahl et al. 1981), and by microbial adherence to solvents (MATS) according to Bellon-Fontaine et al. (1996) with slight modifications. Briefly, untreated and LiCl-treated bacterial cells were washed with and suspended in PUM buffer (22.2 g l^−1^ of potassium phosphate trihydrate, 7.26 g l^−1^ of monobasic potassium phosphate, 1.8 g l^−1^ of urea, and 0.2 g l^−1^ of magnesium sulfate heptahydrate, pH 7.1) to ca. 10^8^ CFU ml^−1^. The cell suspension (5 ml) was mixed with 1 ml of a solvent. Following incubation at 30 °C for 10 min, the mixtures were vortexed vigorously for 2 min and then allowed to stand for 15 min at room temperature to ensure complete separation of the organic and aqueous phases. The absorbance of the aqueous layer was measured at 600 nm. The affinities to the solvent were expressed using the formula: (1 − *A*/*A*_0_) × 100.

### Bacterial adhesion capacity

To assess bacterial adhesion capacity, human colon adenocarcinoma cell line HT29 (ATCC no. HTB-38) was used. Cells were cultured in RPMI 1640 medium supplemented with 10 % fetal calf serum (FCS; Gibco*™*, Paisley, UK) and antibiotics (100 U ml^−1^ penicillin and 100 μg ml^−1^ streptomycin; Sigma, St. Louis, MO) at 37 °C in a humidified atmosphere with 5 % CO_2_. Cells were seeded onto a 24-well tissue culture plate (Nunc, Roskilde, Denmark) at a concentration of 5 × 10^5^ cells ml^−1^. After 24 h of incubation, a monolayer was obtained. Bacterial strains were resuspended in the HT29 growth medium to a final concentration of 5 × 10^7^ cells ml^−1^, and 1 ml of each suspension was added to appropriate wells of the culture plate. After 2 h of incubation, the monolayers were washed three times with phosphate-buffered saline (PBS with Ca^2+^ and Mg^2+^ ions, pH 7.4) to remove bacteria which had not attached to the HT29 cells. Thereafter, the cells were lysed using 0.1 % (v/v) Triton-X100 (Sigma), and the number of viable adherent bacteria was determined by plating serial dilutions on MRS agar plates.

The results of the adhesion assays are expressed as an adhesion index for each strain. The adhesion index is defined as the number of adherent bacterial cells per 100 epithelial cells (Gopal et al. 2001).

### Auto- and co-aggregation assays

The autoaggregation assay was done according to the method of Golowczyc et al. (2007) with a slight modification. Briefly, lactobacilli were harvested at the stationary phase, collected by centrifugation (10,000×*g* for 10 min), washed twice, and resuspended in PBS (pH 7.2). In all experiments, the bacterial suspension was standardized to OD_600_ = 1.0 (2 × 10^8^ CFU ml^−1^). Optical density was measured in a spectrophotometer (Biorad, Germany) at regular intervals (2, 3, 4 and 5 h) without disturbing the microbial suspension, and the kinetics of sedimentation was obtained. The autoaggregation coefficient (AC_*t*_) was calculated at time *t* as:

$$ {\text{AC}}_{t} = \left[ {1 - \frac{{{\text{OD}}_{t} }}{{{\text{OD}}_{i} }}} \right] \times 100 $$

where OD_*i*_ is the initial optical density at 600 nm of the microbial suspension and OD_t_ is the optical density at time *t*.

In the co-aggregation assay, suspensions of lactobacilli were obtained as described previously. Pathogenic bacteria were harvested in the stationary phase by 4-min centrifugation at 5,000×*g* and resuspended in PBS (pH 7.2). One milliliter of *Lactobacillus* suspension and 1 ml of pathogenic bacterial suspension at the same optical density (OD_600_ = 1.0) were mixed. Optical density was measured at regular intervals (2, 3, 4 and 5 h) in order to obtain the kinetics of sedimentation. The co-aggregation coefficient (*CC*_*t*_ *)* was calculated as:

$$ {\text{CC}}_{t} = \frac{{\left[ {\left( {A_{x} + A_{y} } \right)/2} \right] - A_{t} \left( {x + y} \right)}}{{A_{x} + A_{y} /2}} \times 100 $$

### Statistical analysis

The values from all tests performed are the means of three separate experiments ± standard deviation. The data were analyzed using the Excel statistical package. Statistical significances were determined by Student’s *t* test and set at *P * < ψ0.01.

## Results and discussion

**Fig. 1:**
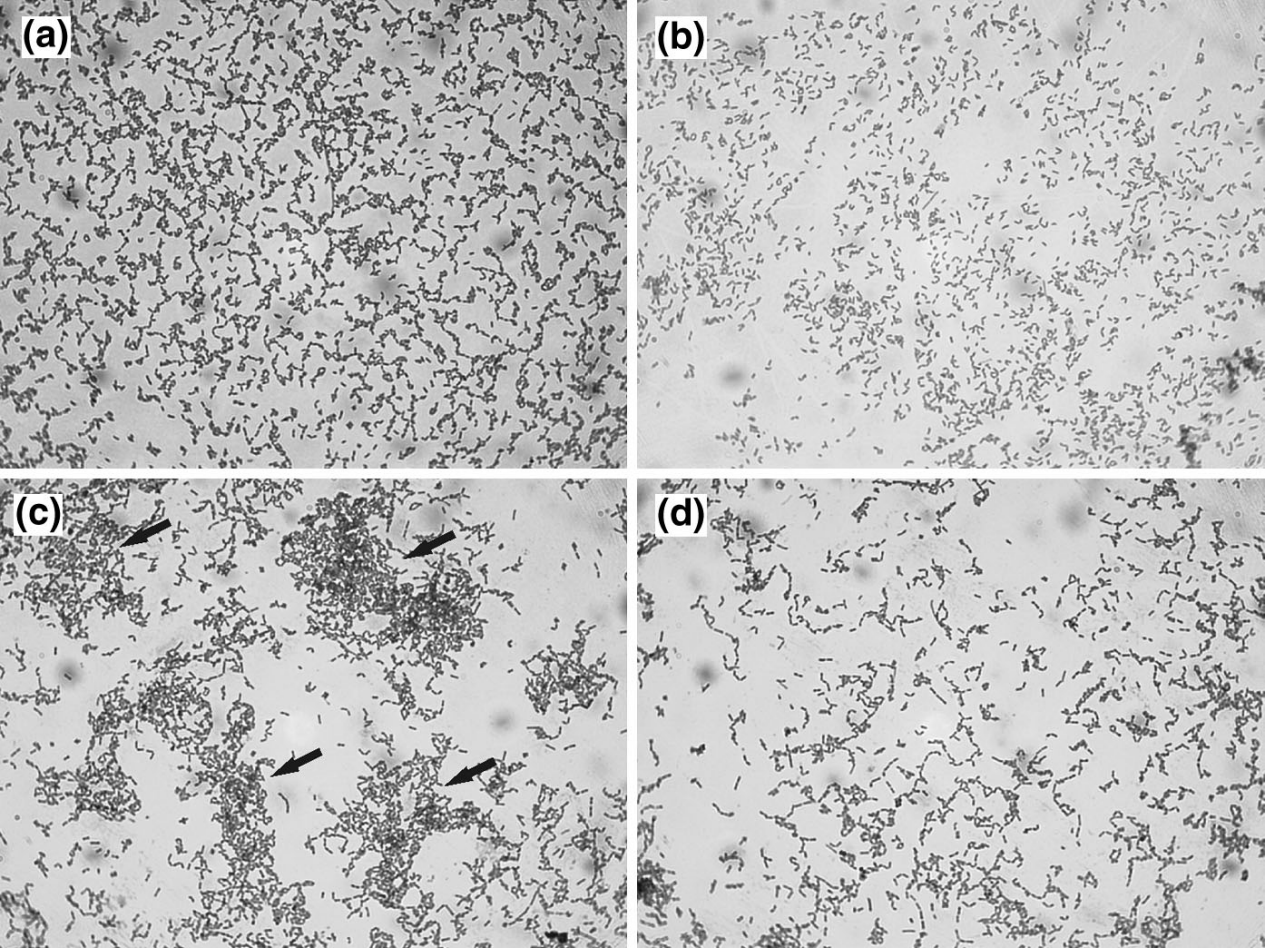
Light microscopy of *Lactobacillus rhamnosus* E/N (**a**, **b**) and PEN (**c**, **d**). Cells suspended in PBS and mixed with ammonium sulfate 0.02 M, pH 6.8 are shown in **a** and **c**, *arrows* indicate large cells aggregates. Untreated cells which served as controls are shown in figures **b** and **d** (×1,000)

In the present study, the SAT and MATS methods were used to investigate the hydrophobic/hydrophilic and Lewis acid–base properties of bacterial surfaces. Both *Lactobacillus* strains tested formed cell aggregates after 1 min at a salt concentration of 0.02 M. It is notable that *L. rhamnosus* PEN formed much larger aggregates than *L. rhamnosus* E/N in the same experimental conditions (Fig. 1). As shown in Table 1, both strains had a strong affinity for chloroform and ethyl acetate as well as low adherence to hexadecane. After LiCl treatment, the adherence to the solvents decreased significantly. Cells devoid of surface proteins did not adhere to hexadecane. The low percentage of bacteria adhering to a nonpolar solvent such as hexadecane indicated that the strains tested had low hydrophobicity. Both strains displayed a higher affinity for acidic solvents, such as chloroform, and a lower affinity for basic solvents, such as ethyl acetate. These results demonstrate that lactobacilli are strong electron donors and weak electron acceptors, as confirmed by their hydrophilic cell surface properties. In other words, the strains tested in the present study have a strong basic and a weak acidic character. The quantitatively important existence of chemical groups such as –COO^−^ and –HSO_3_^−^ on the surface of the microorganisms could explain their strong electron donor character (Pelletier et al. 1997). The hydrophilic nature of lactobacilli has also been demonstrated in previous studies (Begovic et al. 2010; Deepika et al. 2009; Gong et al. 2012).

**Table 1:** Adhesion of untreated and LiCL-treated cells of *L. rhamnosus* PEN and E/N to chloroform, hexadecane, and ethyl acetate

Bacteria	Adhesion (%)		
	Chloroform	Ethyl acetate	Hexadecane
*L. rhamnosus* PEN, untreated	81.62 ± 0.006	53.83 ± 0.02	11.77 ± 0.01
*L. rhamnosus* PEN, LiCl-treated	62.23 ± 0.006	36.72 ± 0.06	nd
*L. rhamnosus* E/N, untreated	76.49 ± 0.002	39.03 ± 0.006	16.78 ± 0.003
*L. rhamnosus* E/N, LiCl-treated	19.78 ± 0.07	2.88 ± 0.006	nd

*nd* not detected

**Fig. 2:**
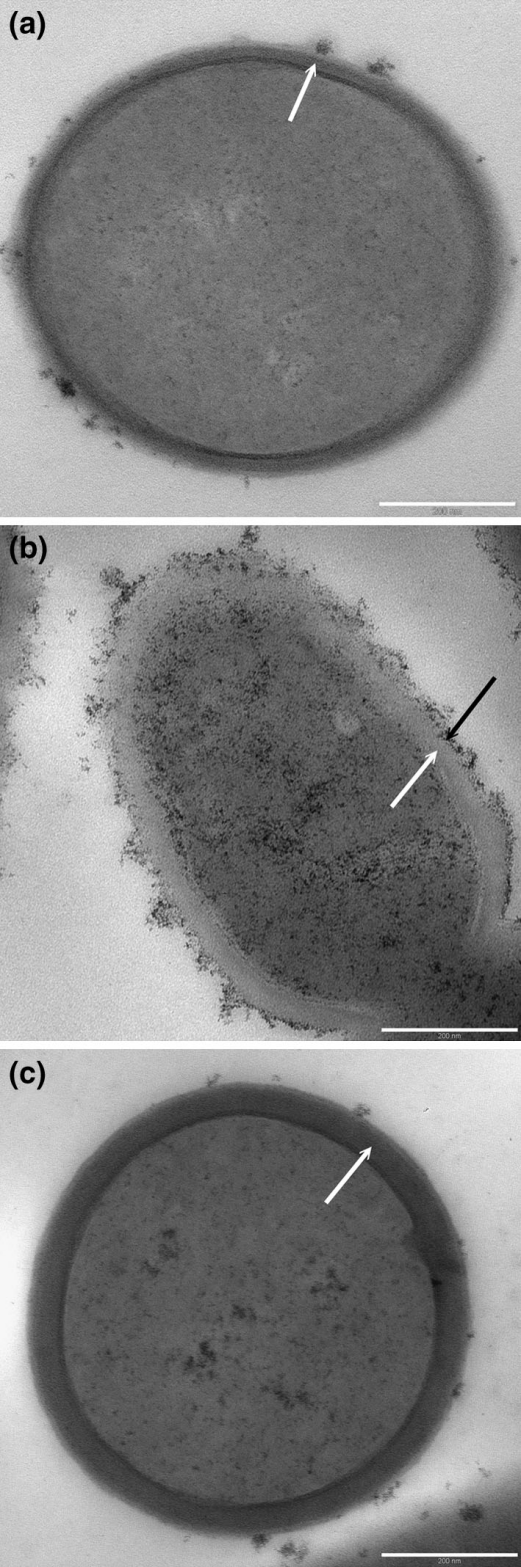
TEM micrographs of *L. rhamnosus* DSMZ 20021 as a negative control (**a**), *L. rhamnosus* E/N (**b**) and *L. rhamnosus* PEN (**c**). *Arrows* indicate the bacterial cell wall (*white arrow*) and EPS layer (*black arrow*)

It is shown in this study that the phenotype differences between the examined strains were related to biosynthesis of cell proteins and EPS. In addition, the capability of *L. rhamnosus* E/N strain for efficient biosynthesis of EPS has also been shown in a previous study (Polak-Berecka et al. 2013a). Differences in bacterial colony morphology, as seen with the naked eye, were observed when the strains were grown on MRS agar medium. When cultured on agar plates, *L. rhamnosus* E/N formed ropy colonies with a glistening and slimy appearance which were able to produce strands. The highest degree of slime production was observed in the liquid culture of *L. rhamnosus* E/N (+++ on the semi-quantitative scale by Christensen et al. 1985). A complete loss of colony mucoidity and slime production was observed for *L. rhamnosus* PEN. Further visualization by TEM revealed a clear cell wall with fibrillar materials on the bacterial surface, consistent with the formation of the extracellular polysaccharides by *L. rhamnosus* E/N (Fig. 2).

**Fig. 3:**
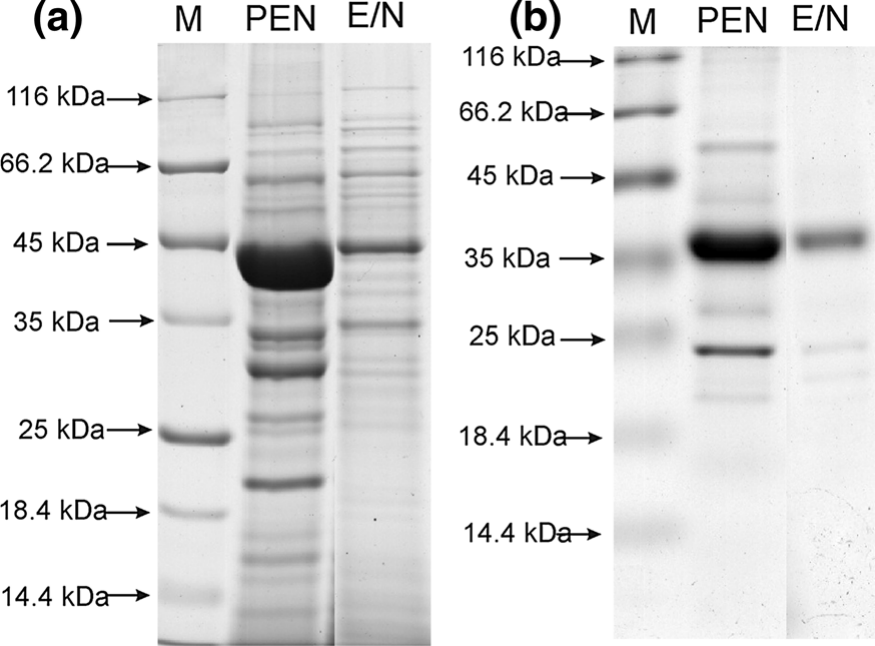
SDS-PAGE analysis of proteins from *L. rhamnosus* PEN and E/N **a** whole cells **b** cell surface proteins. *M* molecular mass marker

Gels from the SDS-PAGE analysis of whole cell proteins and cell surface proteins of *L. rhamnosus* PEN and E/N are shown in Fig. 3. The analysis of whole cell proteins indicated that the number of bands was similar in both strains, with molecular weights (MW) ranging between 13.8 and 207 kDa. Some differences were observed in the case of low molecular mass proteins. In *L. rhamnosus* PEN, additional, small MW proteins from 13 to 30 kDa were detected, while in *L. rhamnosus* E/N, a greater amount of high MW proteins was observed (45–207 kDa). After LiCl treatment, we observed that the examined strains had a low amount of non-covalently bound proteins, as seen in Fig. 3b. However, the band patterns changed significantly between the tested strains. The protein profiles differed in terms of the molecular mass and number of detected bands. In *L. rhamnosus* PEN, the number of bands was almost three times greater than in *L. rhamnosus* E/N. This difference resulted from the presence of high MW proteins, between 42 and 114 kDa, in the *L. rhamnosus* PEN surface protein profile, not observed in *L. rhamnosus* E/N. Other authors have reported that proteins of a similar size have been implicated in contributing to adhesive properties of lactobacilli (Ramiah et al. 2008; Macías-Rodríguez et al. 2009). These include 45 and 58 kDa proteins that bind to collagen type I (Lorca et al. 2002) and a 64 kDa fibronectin binding protein (Munõz-Provencio et al. 2010).

**Fig. 4:**
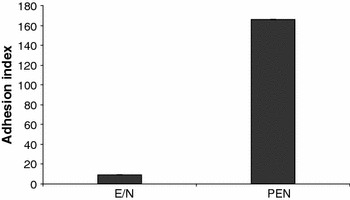
In vitro adhesion of *Lactobacillus rhamnosus* E/N and PEN to HT-29 cells. Results are mean from three independent experiments. Standard deviations were ±0.1 for *L. rhamnosus* E/N and ±0.3 for *L. rhamnosus* PEN

Physicochemical factors have so far been recognized as nonspecific mechanisms that play an important role in bacterial adhesion (Greene and Klaenhammer 1994). If, however, the lactobacilli used in this study adhere by a nonspecific mechanism, the similar hydrophilic character of these strains would suggest that they should have quantitatively similar (presumably low) attachment to human colon adenocarcinoma cell line HT29. However, the present study revealed a significant difference in the values of the adhesion index between the strains tested (Fig. 4). High adhesion was observed for *L. rhamnosus* PEN, with *A*_*x*_ = 166 (8.3 × 10^5^ CFU ml^−1^ adherent cells). By contrast, *L. rhamnosus* E/N showed a significantly reduced adhesion capacity, with *A*_*x*_ = 9 (4.5 × 10^4^ CFU ml^−1^ adherent cells). This finding is in agreement with previous reports, which have indicated that adhesion is strain-dependent (Jensen et al. 2012) and is determined by specific adhesion mechanisms. Gopal et al. (2001) determined an adhesive index *A*_*x*_ = 105 for probiotic *L. rhamnosus* GG to HT 29 cells. The authors observed for other probiotic strains *A*_*x*_ values up to this level and described them as strains with high adhesion ability. The fact that a large number of *L. rhamnosus* PEN cells adhered to the monolayer of HT29 cells suggested that adhesion was facilitated by cell surface-bound proteins. It seems that the low adherence ability of *L. rhamnosus* E/N to HT29 cells was due to the EPS envelope covering the producing strains. Both CPS and EPS hinder bacterial adhesion by shielding proteins and other surface molecules that could act as adhesins. Nikolic et al. (2012) also observed that the presence of an EPS layer surrounding *Lb. paraplantarum* hindered its adhesion to the epithelial intestinal cell lines Caco-2 and HT29. Horn et al. (2013) reported that a decrease in EPS production in *Lb. johnsonii* led to a large increase in autoaggregation and adhesion of these bacteria to HT29 cells. EPS production, however, is not the only factor that determines bacterial adherence. The polymer size and the specific chemical structure and composition of the EPS molecule could affect adhesion as well. Lebeer et al. (2009) observed a concomitant increase in biofilm formation and adhesion to Caco-2 monolayers for *L. rhamnosus* GG that had lost the ability to produce large-size galactose-rich EPS and produced only small-size glucose-rich cell-wall associated polysaccharides. Similarly, in the present study, a low adherence ability was observed in *L. rhamnosus* E/N, which produces rhamnose-rich EPS (Polak-Berecka et al. 2013b). It has been reported that mutations in the *eps* gene cluster affect aggregation as well as adhesion. Aggregation may affect both colonization and interactions with other bacteria (Lebeer et al. 2008; Walter et al. 2008). However, in some cases, EPS could be a positive mediator of bacterial adhesion (Walter et al. 2008; Sims et al. 2011). The chemical composition of EPS determines its interactions with other surface factors. For example, glucans play an important role in biofilm formation in the human oral cavity (Sims et al. 2011).

**Fig. 5:**
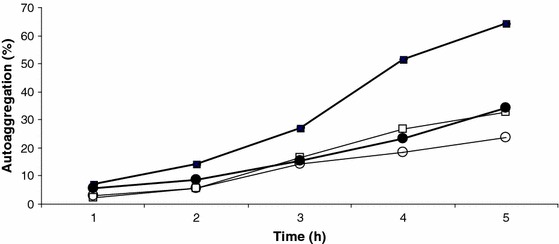
Autoaggregation percentages of *L. rhamnosus* PEN (*black square*) and *L. rhamnosus* E/N (*black circle*) cells. *Closed* and *open* symbols represent untreated and LiCl-treated cells, respectively. Each *value* represents an average of triplicate measurements; standard deviations were ±0.1–0.2 (for untreated PEN cells), ±0.1–0.3 (for LiCl-treated PEN cells), ±0.06–0.3 (for untreated E/N cells), and ±0.1–0.2 (for LiCl-treated E/N cells)

**Fig. 6:**
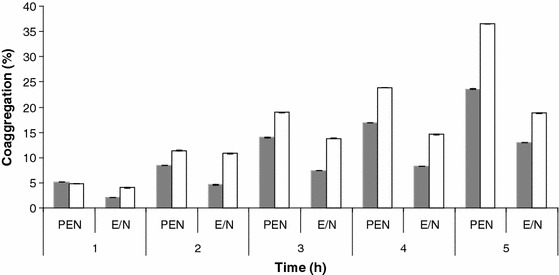
Coaggregation of *L. rhamnosus* PEN and *L. rhamnosus* E/N with *Salmonella anatum* (*grey bars*) and *Staphylococcus aureus* (*white bars*). The *graph* shows the mean values (from three independent experiments) with standard deviation in the range 0.01–0.06

Lactobacilli that adhere well to epithelial cells have also been reported to have a high aggregation ability (Nikolic et al. 2010). Strains expressing fast autoaggregation ability have exhibited a high percent of adhesion to chloroform and ethyl acetate. Similar results were observed with *L. rhamnosus* PEN. Moreover, in this strain, the high autoaggregation ability (Fig. 5) and coaggregation with *Salmonella anatum* and *Staphylococcus aureus* (Fig. 6) was influenced by surface proteins. After LiCl treatment, the ability of this strain to form aggregates significantly decreased. It is also possible that, besides or together with proteins, the aggregation process involves some ions (Nikolic et al. 2010). *L. rhamnosus* E/N had a significantly reduced ability to aggregate, possibly because of the absence of proteinaceous factors responsible for auto- and co-aggregation. Moreover, in this strain, EPS shielded alternative ligands that reside in the bacterial cell envelope (Lee et al. 2013).

**Fig. 7:**
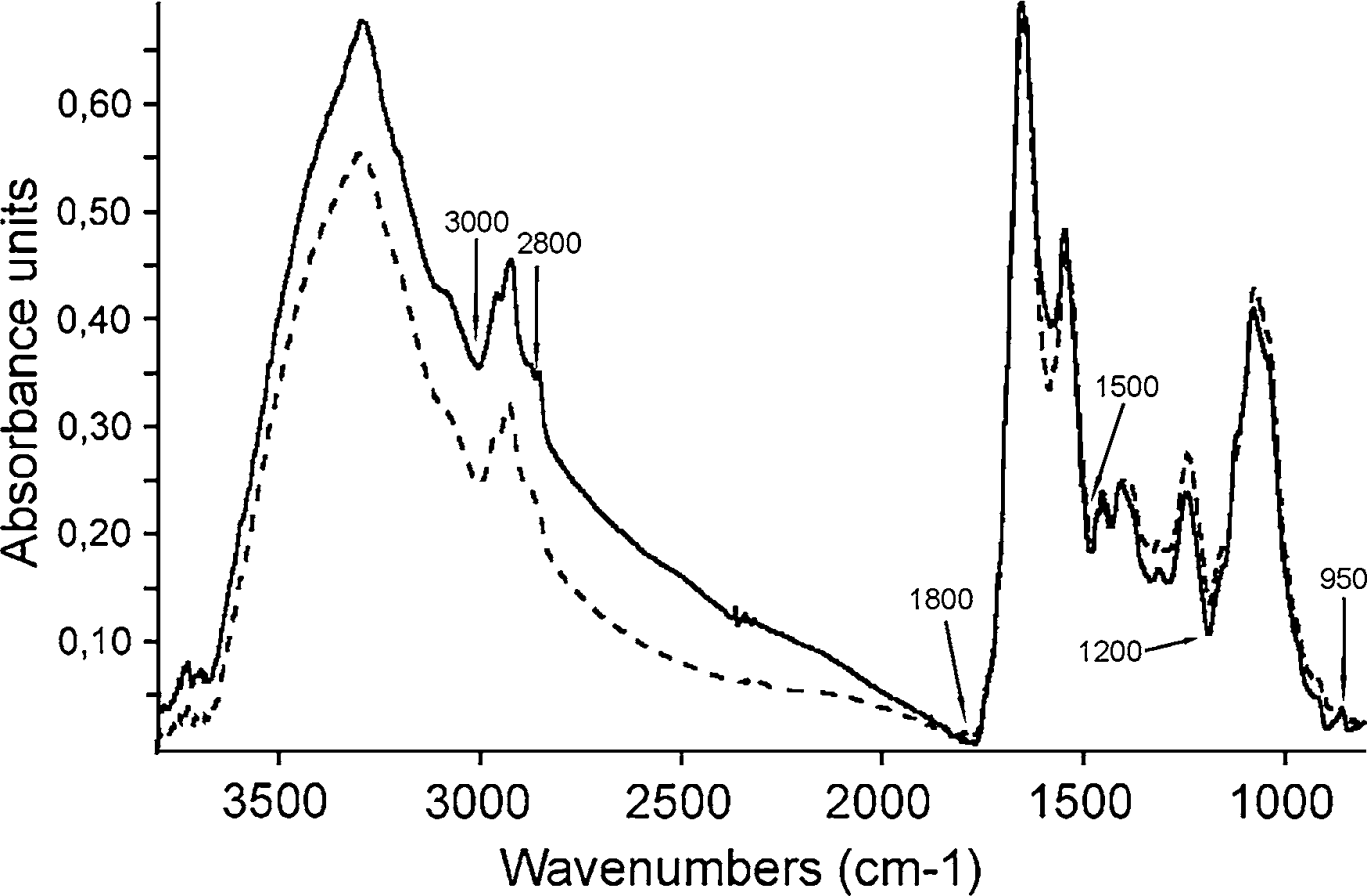
FT-IR spectra of freeze-dried bacteria: *solid line*—*L. rhamnosus* E/N and *broken line*—*L. rhamnosus* PEN. The spectra were averaged, normalized, and *baseline* corrected in the 3,800–800 cm^−1^ range

In this work, we used FT-IR to complement the data from hydrophobicity and SDS-PAGE analyses. The greatest differences among the samples were observed in the fatty acid regions (3,000–2,800 cm^−1^). Slight peak shifts were visible in the amide region, dominated by amides I and II of various peptides (1,800–1,500 cm^−1^), in a mixed region of fatty acid bending vibrations, proteins, and phosphate-carrying compounds (1,500–1,200 cm^−1^), and in areas corresponding to the carbohydrate region (1,200–950 cm^−1^) of the spectra (Fig. 7). In both strains, absorption bands emerged at 1,400 cm^−1^, which came from COO^−^ groups of amino acids and fatty acids. The analysis of the data presented in Table 1 indicates that *L. rhamnosus* E/N had a slightly weaker basic character compared to PEN. This can be explained by the fact that the cells of this strain are covered by a layer of both neutral and acidic polysaccharides, which can be either cell-wall-associated or extracellular (Schär-Zammaretti and Ubbink 2003; Polak-Berecka et al. 2013b). FT-IR data (1,200–950 cm^−1^) showed differences between the two strains in the vibrations of C–OH and C–O-C groups dominating in various polysaccharides. In most lactobacilli, high cell surface hydrophobicity is determined by basic S-layer proteins. However, no S-layer has been reported for *L. rhamnosus* species. Thus, the surface properties of *L. rhamnosus* PEN could be determined by covalently anchored proteins. The N-terminally anchored proteins represent the largest group of cell-surface-anchored proteins in lactobacilli and are mainly involved in cell-envelope metabolism, extracellular transport and signal transduction, competence, and protein turnover (Sengupta et al. 2013). In our work, FT-IR analyses showed differences in the absorption band of 1,544 cm^−1^, which indicated that the vibration came from N–H bending and C–N stretching in amides II. In our study, the treatment of the bacteria with LiCl did not significantly change the character of the cell surface, suggesting that the proteins were covalently bound, and thus they were most likely not S-layer proteins. After LiCl treatment, *L. rhamnosus* E/N showed a more hydrophobic character than the PEN strain. This may indicate that the EPS layer of *L. rhamnosus* E/N was removed together with surface proteins loosely attached to the cell. The notable difference in bacterial affinity for hexadecane and chloroform, solvents with identical van der Waals forces, showed the importance of the Lewis acid–base interactions on the cell surface. These data demonstrated the capacity of the *Lactobacillus* strains tested to establish some interactions by mechanisms other than van der Waals forces (Pelletier et al. 1997). The adhesive properties of LAB include different features such as passive forces, electrostatic interactions, hydrophobic steric forces, lipoteichoic acids and lectins (Nikolic et al. 2010). The FT-IR analysis revealed that the investigated *L. rhamnosus* strains differed significantly in the 3,000–2,800 cm^−1^ region corresponding to the C–H asymmetric stretching of –CH_3_ and >CH_2_ groups in fatty acids. This observation is in line with FAME data, which demonstrated a difference between the tested strains in the saturated/unsaturated ratio and the average chain length of the fatty acids. In *L. rhamnosus* E/N, unsaturated fatty acids accounted for 70.5 % and saturated fatty acids for 29.5 %, and for 41.2 and 58.8 %, respectively, in *L. rhamnosus* PEN (Table 2). The cellular fatty acid composition, especially the saturated/unsaturated fatty acid ratio, plays an important role in membrane stability and permeability by increasing the fluidity of the cytoplasmic membrane (Gong et al. 2012). However, the specific role of membrane fatty acids in terms of bacterial adhesion ability is still poorly understood. Overall, FT-IR supported the earlier observation that the surfaces of the tested lactobacilli differed with respect to the carbohydrate and protein composition, and confirmed the unique fatty acid content of these strains. It has previously been demonstrated that the fatty acid composition of bacterial lipids has a profound effect on the adhesion ability of bacterial strains (Kankaanpää et al. 2004). In our study, the relationship observed between the different fatty acid compositions and the different adhesion abilities of the tested strains is consistent with previous reports. The fatty acid composition of lactobacilli may largely influence other factors associated with the microbial adhesion process, probably by affecting bacterial membrane fluidity and membrane-lipopeptide interactions (Gusils et al. 2002).

**Table 2:** Membrane fatty acid composition of *L. rhamnosus* E/N and PEN

Membrane fatty acid	*L. rhamnosus* E/N	*L. rhamnosus* PEN
	%	%
Lauric C12:0	nd	0.0076 ± 0.0001
Myristic C14:0	0.0124 ± 0.0002	0.0151 ± 0.0002
Palmitic C16:0	0.0361 ± 0.0003	0.0871 ± 0.001
Oleic C18:1n9c	0.1140 ± 0.0018	0.0645 ± 0.0011
Linolelaidic C18:2n6t	0.0429 ± 0.0006	0.0227 ± 0.0003
Elaidic C18:1n9t	0.0165 ± 0.0001	0.0285 ± 0.0002
Stearic C18:0	0.0240 ± 0.0002	0.0553 ± 0.0008
Total saturated	0.0725	0.1652
Total unsaturated	0.1734	0.1157
Ratio saturated/unsaturated	0.4183	1.4270

*nd* not detected

It can be concluded from our comparative analysis that the hydrophobic nature of the bacterial surface cannot be taken as a reliable indicator of adhesion or aggregation in vitro. The present study may contribute to explaining this fact, as aggregation and adhesion ability can be conditioned by a multitude of factors (protein factors, hydrophobicity, fatty acids, and EPS) that may positively or negatively affect adherence to other cells. In the investigated *L. rhamnosus* strains, adherence to HT29 or to other bacteria was strongly dependent on specific interactions and was promoted by surface proteins of MW between 42 and 114 kDa. Presumably, the saturated/unsaturated fatty acid ratio played an important role in promoting adhesion. Polysaccharides, on the other hand, hindered adhesion and aggregation by masking protein receptors. At the same time, these specific molecules were responsible for the hydrophilic nature of the two strains tested.
